# Targeting Protein Kinase C Pathways With Endoxifen for Impulsivity and Behavioural Dysregulation in an Adolescent With Intellectual Disability: A Case Report

**DOI:** 10.1192/bjo.2026.11940

**Published:** 2026-06-30

**Authors:** Pooja Sharma

**Affiliations:** Neuromind Clinic, Delhi, India

## Abstract

**Aims:**

Inattention and hyperactivity/impulsivity are frequently associated with intellectual disability (ID), contributing to significant behavioural dysregulation and functional impairment. Protein kinase C (PKC) has emerged as a key molecular target in the neurobiology of impulsivity, with PKC overactivity disrupting dopaminergic regulation and prefrontal cortical functioning, leading to impaired inhibition and executive dysfunction. Endoxifen, an active metabolite of Tamoxifen, is a selective PKC inhibitor that has demonstrated antimanic, anti-impulsive, and emotion-stabilising effects in adults. Emerging clinical evidence suggests benefit in behavioural dysregulation across several psychiatric conditions; however, paediatric use remains limited and largely off-label. This case report aims to evaluate the efficacy and tolerability of endoxifen for behavioural symptoms in an adolescent with ID who showed inadequate response to standard treatments.

**Methods:**

A 14-year-old male with global developmental delay and significant speech–language impairment presented with repetitive verbal behaviours, hyperactivity, poor sitting tolerance, and frequent anger outbursts for two years. He was provisionally diagnosed with ID with behavioural impairment. Baseline BMI was 21 kg/m², and comorbid hypothyroidism was managed with replacement therapy.

Previous trials of risperidone (4 mg/day) with divalproex sodium (1500 mg/day) resulted in minimal improvement and adverse effects, including nausea, increased appetite, and weight gain. Subsequent treatment with oxcarbazepine (titrated to 1350 mg/day) and aripiprazole (10 mg/day) was complicated by hyponatremia, necessitating discontinuation of oxcarbazepine.

Following informed consent and assent, endoxifen was initiated at 8 mg/day and gradually titrated to 24 mg/day, while aripiprazole was continued at 5 mg/day. Clinical response and tolerability were monitored over 12 months.

**Results:**

Over one year of treatment, the patient demonstrated sustained reductions in impulsivity, hyperactivity, repetitive behaviours, and anger outbursts, with notable improvements in behavioural regulation and overall family functioning. The medication was well tolerated, with no significant metabolic, neurological, or systemic adverse effects, and adherence remained satisfactory throughout follow-up.

**Conclusion:**

Endoxifen showed favourable efficacy and tolerability in managing behavioural dysregulation in an adolescent with ID who was refractory or intolerant to conventional psychotropics. Targeting PKC-mediated pathways may represent a promising therapeutic strategy for impulsivity and aggression in neurodevelopmental disorders. Larger controlled studies are needed to establish safety and long-term efficacy.

